# Bibliography

**Published:** 1902-11-15

**Authors:** 


### BIBLIOGRAPHY.

"ANATOMY AND HISTOLOGY OF THE MOUTH AND TEETH." The second edition of Broomell's "Anatomy and Histology of the Mouth and Teeth'' has just come from the press of P. Blakiston's Son & Co. As a specimen of the book makers art is a most creditable example. It is printed on heavy sized paper and the cuts show up with a distinctness that is entirely satisfactory, we seldom find a work so satisfactorily done from this standpoint. The work is substantially a reprint of the first edition, with the correcton of some errors in the former, and the addition of about fifty new illustrations, and a chapter on embryology and another on anomalies of tooth form and structure. All that the author has attempted he has well done. We wish he could have given more space to the embryology and histology of the regions of such great interest to the dentist. There is no work which treats this subject in as comprehension a manner as its importance deserves. The author makes a kind of apology for allowing the portion devoted to regional anatomy to remain in spite of adverse criticism. We are of the opinion that not only should this be retained, but that it would be much better had the author made use of some of the work done by Prof. Cryer. This feature could well be elaborated. The work on tooth anatomy is well done and the illustrations are very good indeed. Altogether it is one of the most satisfactory books on any phase of dental science.

